# Sexual orientation differences in mental health service use and unmet mental health care needs: a cross-sectional population-based study of young adults

**DOI:** 10.1007/s00127-025-02866-8

**Published:** 2025-03-11

**Authors:** Luis Roxo, John Pachankis, Richard Bränström

**Affiliations:** 1 Division of Psychology, Department of Clinical Neuroscience, Karolinska Institutet, Stockholm, Sweden; 2 Department of Social and Behavioral Sciences, Yale School of Public Health, New Haven, CT USA; 3 Comprehensive Health Research Centre (CHRC), Universidade NOVA de Lisboa, Lisbon, Portugal

**Keywords:** Disparities, LGBT, Mental health services, Sexual orientation, Unmet needs

## Abstract

**Purpose:**

This study aims to examine sexual orientation differences in mental health services use and unmet mental health care needs, and to explore associated sociodemographic factors in a populational-based sample of Swedish young adults (aged 18–34).

**Methods:**

Data from the Pathways to Longitudinally Understanding Stress (PLUS) study were used (2019, *N* = 2,126, participation rate of 37.8%). We performed logistic regressions to model mental health services use and unmet mental health service needs, followed by a subgroup analysis among those with perceived need for mental health services. We also examined if associations between sociodemographic factors (gender, age, educational level, income, employment status, household composition, urbanicity and country of birth) and these outcomes varied by sexual orientation.

**Results:**

Around one-third (35.0%) of sexual minority individuals had used mental health services the previous year, versus 20.2% of heterosexuals (OR = 1.52, 95%CI = 1.17–1.96, *p* = 0.002). Unmet needs were more likely among sexual minority individuals (17.6%) than heterosexuals (11.8%, OR = 1.47, 95%CI = 1.09-2.00, *p* = 0.013), with no significant sexual orientation differences among participants perceiving a need for mental health services. Among those with perceived need for mental health services, it was estimated that 56% of sexual minority males had unmet needs (vs. 37% of heterosexual); no such difference existed among women. Other than gender, there were not significant interactions between sociodemographic factors and sexual orientation.

**Conclusion:**

Sexual minority individuals’ higher mental health services use highlights the need for high-quality, culturally sensitive services. Future research should identify reasons for the greater proportion of unmet mental health service needs among sexual minority men.

**Supplementary Information:**

The online version contains supplementary material available at 10.1007/s00127-025-02866-8.

## Background

Sexual minority (i.e., lesbian, gay, bisexual [LGB], and other non-heterosexual) individuals are more likely to experience poor mental health, and an increased prevalence of common mental disorders (e.g., depression and anxiety) and suicidal ideation compared to heterosexuals [1–3]. Studies suggest that this increased risk is at least partially explained by exposure to social stress, such as sexual minority individuals’ higher likelihood of experiencing discrimination, victimization, and threats to social safety over the life course [1, 4, 5]. Likely as a result of this increased risk for common mental disorders, sexual minority individuals are also more likely to use mental health services and receive mental health treatment compared to heterosexuals [1, 2, 6–8].

Even though sexual minority individuals are more likely to be treated for mental health diagnoses compared to heterosexuals, studies have shown that unmet mental health care needs (often defined as not using mental health services despite a need for mental health treatment) are high among sexual minority individuals [7, 9–11]. Yet, there is great variation between studies, from 42.9% in an adult US sample to more than two-thirds among a sample of sexual minority women recruited on the internet [11, 12]. Studies reporting unmet mental health care needs among sexual minority individuals typically come from online surveys administered to non-representative samples; in fact, research on this topic rarely uses populational-based probability samples. Unmet mental health care needs are also measured in different ways across studies. Some studies define unmet mental health care needs as the proportion of individuals reporting elevated levels of mental health symptoms that do not receive treatment [9, 11, 13, 14], and other studies define unmet mental health care needs as the proportion of individuals reporting a self-perceived need for services that is unfulfilled [14, 15]. The latter approach assumes that a self-perceived need reflects one’s evaluation of symptoms, distress, functioning, and ability to manage one’s well-being, regardless of their elevated mental health symptoms [14].

It is also unclear if sexual minority individuals are more or less likely to experience unmet mental health care needs than heterosexuals. A small number of previous studies have shown that sexual minority individuals experience more frequent unmet mental health care needs than heterosexuals [12, 16], but other studies have found no such pattern [17]. Some studies have even found the opposite pattern, namely that sexual minority individuals experience less unmet mental health care needs than heterosexuals [7–9, 13, 18]. Still, other studies have found higher unmet mental health care needs only among some subgroups of sexual minorities (e.g., higher unmet mental health care needs among sexual minority women compared to heterosexual women, but no such sexual orientation pattern among men) [15]. These inconsistent findings likely result from the large variation in study samples, methodologies, and definitions of unmet mental health care needs. Also, most studies examining unmet mental health care needs are from the US [19], where access to mental health services is heavily influenced by health care insurance coverage and socioeconomic status; therefore, it is unclear how these results might generalize to other countries.

Several sociodemographic factors have been linked to unmet mental health care needs, including gender, age, and educational level. Studies have shown that women, those with more education, and middle-aged persons are more likely to use mental health services and less likely to experience unmet mental health care needs compared to men, those with lower education, and younger and older people [20–22]. However, it is largely unknown if sociodemographic differences in mental health service use and unmet mental health care needs vary by sexual orientation [7]. Studies have suggested that sexual minority status intersects with other social identities to sometimes synergistically generate risk that cannot be predicted by either identity alone [19, 23, 24]. Thus, it is plausible that the impact of sociodemographic variables differs between sexual minority individuals and heterosexuals.

In the current study, we aimed to overcome some of the limitations of previous research (e.g., predominant use of internet samples, lack of evidence from countries other than the US), by using a population-based sample of young adults from Sweden to examine sexual orientation differences in mental health services use and unmet mental health care needs. The context of Sweden is particularly suitable for studying unmet mental health care needs among sexual minority individuals given that the Swedish universal health care system eliminates potential confounding due to sexual orientation differences in health insurance which have been observed in the US [25, 26], and aims to provide mental health care based on need [20]. Young adulthood is a particularly important age period for the study of unmet mental health care needs since this life stage represents a sensitive period for the development of most mental disorders [26]. Previous studies have also shown that sexual orientation disparities in mental health symptoms and diagnoses are present early in life [27]. Moreover, previous evidence from Sweden has shown that 44% of young adults who perceive a need for mental health treatment do not seek such services, a proportion that is higher than other age groups [20].

The first aim in this study was to examine sexual orientation differences in mental health service use and unmet mental health care needs (defined as not receiving mental health treatment despite a perceived need for such services). Based on the existing literature, we hypothesized that: H1) Sexual minority individuals use mental health services more often than heterosexuals; and H2) sexual minority individuals have a higher proportion of unmet mental health care needs, compared to heterosexuals. The corresponding null hypotheses are: H0a: Mental health services use is equally likely among sexual minority individuals and heterosexuals; and H0b: Unmet mental health care needs are equally likely among sexual minority individuals and heterosexuals. As a secondary objective, we explored sociodemographic differences in mental health services use and unmet mental health care needs and whether these differences vary by sexual orientation. This exploratory analysis examined the relationships between gender, age groups, educational level, employment status, income, relationship status, urbanicity and country of birth with the outcome variables.

## Methods

### Study sample

We used data from the first wave of the Pathways to Longitudinally Understanding Stress (PLUS) cohort study. Participants in the PLUS cohort were recruited from the Swedish National Public Health Survey (SNPHS). Specifically, during Fall 2019, all young adults (ages 18–34) who identified as non-heterosexual in the 2015, 2016, and 2018 SNPHS were invited to participate, along with an equally sized randomly selected sample of heterosexual participants from the SNPHS. A total of 5,885 participants were contacted and 2,222 (participation rate of 37.8%) provided written informed consent and completed the PLUS Wave 1 online survey assessment. Sampling weights were calculated to adjust for non-response and to reflect the age and sex distribution of the Swedish young adult population, thus adjusting the sample to be more representative of the population. Our final analytic sample consisted of the participants who successfully completed all self-report measures included in the analyses for the current study, a total of 2,126 (flowchart available in Online Resource 1).

### Measures

#### Sexual orientation

Several questions were used to assess sexual orientation: (1) “*Which of the following best represents how you think of yourself?*” with the response options “*lesbian or gay*;” “*straight*,* that is*,* not lesbian or gay*;” “*bisexual*;” “*something else*;” or “*I don’t know the answer*.” Those participants answering “*something else*” were asked a second question: (2) “*What do you mean by something else?*” with the response options being “*queer*;” “*pansexual*;” “*asexual*;” “*demisexual*;” and “*none of the above*.” Participants responding “*none of the above*” were then asked (3): “*What term or terms do you use to identify your sexual orientation?*” with an open text response, and the subsequent question: (4) “*This study is about individuals who are not heterosexual/straight and so asks about people’s experiences being LGBTQ+. You indicated that you identify as* [participant’s response to question (3)]. *Are you comfortable being referred to as LGBTQ + throughout this survey?*” with the response options “*yes*” or “*no*.” Participants were classified as sexual minority individuals if they reported identifying as: lesbian, gay, bisexual, queer, pansexual, asexual, demisexual, or otherwise confirmed that they were comfortable being referred to as LGBTQ+.

#### Perceived need for mental health services

Participants’ perceived need for mental health services was assessed with the question “*Was there ever a time during the past 12 months when you felt that you might need to see a professional because of problems with your emotions*,* nerves*,* or your use of alcohol or drugs?*” with the response categories “*yes*” and “*no.*” Based on the responses to the question, participants were categorized into those who perceived a need for mental health services and those who did not perceive a need for mental health services. Previous research has shown an association between perceiving a need for mental health services and having serious mental health symptoms, supporting the use of this self-assessment [14].

#### Mental health services use

Those reporting a need for mental health services were subsequently asked if they had visited a professional for help with their mental health concerns during the past 12 months: “*In the past 12 months*,* did you go to see any of the professionals on this list for problems with your emotions*,* nerves*,* or your use of alcohol or drugs?*” Participants were considered as having used mental health services when they reported having visited a professional for mental health support during the previous 12 months.

#### Unmet mental health care needs

Participants were categorized as experiencing unmet mental health care needs if they perceived a need for mental health services but had not seen any professional for their mental health concerns during the past 12 months [14]. This information was used to calculate two measures: (a) proportion of individuals with unmet mental health care needs among the whole sample; and (b) proportion of individuals with unmet mental health care needs among those reporting a perceived need for mental health treatment.

#### Sociodemographic covariates

Gender was coded as *male* (those reporting their current gender identity as *“male”* or *“trans male/trans man”*), *female* (*“female”* or *“trans female/trans woman”*), or *other gender identity* (*“genderqueer/gender non-conforming”* or *“different identity”*). Age was categorized as *18–24* or *25–34.* Educational level was coded as *low* (high school or less), *moderate* (some college or technical school), and *high* (university degree or higher). Monthly income in the previous year was coded as *less than 10*,*000 SEK/month*,* 10*,*000–29*,*999 SEK/month*, and *30*,*000 SEK/month or more.* Those working full- or part-time were categorized as *employed*, while unemployed individuals, non-working students and participants with a disability were considered *not employed.* As a measure of household composition, we classified participants who reported being in a relationship and living with their partner (whether married or not) as *living with a partner*. We categorized participants who were living in towns, villages, or unincorporated areas as *living in a rural area*, while those residing in cities with over 10,000 inhabitants were categorized as *living in an urban area*. Place of birth was coded as *Sweden* or *other country*.

### Statistical analysis

First, we examined sociodemographic differences by sexual orientation, using descriptive statistics. To pursue our first aim, we analyzed sexual orientation differences in mental health services use and unmet mental health care needs using logistic regressions. As stated above, we calculated two measures of unmet mental health care needs: the proportion of the total sample that perceived a need and did not use services (to explore sexual orientation differences in the proportion of people with unmet mental health care needs), and a subgroup analysis only among those with perceived need of health care (to assess sexual orientation differences in help-seeking behaviors among those with a perceived need for mental health services). In all analyses, we first used unadjusted models, and then, models adjusted for sociodemographic covariates (i.e., gender, age, educational level, employment status, income, relationship status, urbanicity, and country of birth).

To pursue our second aim, we first calculated the significance of the association between sociodemographic variables and the dependent variables. Next, we included the interaction between sexual minority status and each sociodemographic covariate, to understand if sexual orientation differences in mental health services use and unmet mental health care needs differed by sociodemographic factors. Then, a multivariate model was computed for each outcome, retaining only significant (< 0.05) main effects and interactions. At this stage, participants reporting “other gender identity” (*n* = 25) were excluded, due to the low number of cases and an uneven distribution between sexual minority individuals and heterosexuals. Odds ratios (OR) and 95% confidence intervals (CI) are presented to interpret the magnitude and significance of the results. The “estimated means” option from the GENLIN function in SPSS was used to compute expected proportions by sociodemographic variables. Statistical analyses were performed using SPSS version 28.

## Results

### Descriptive statistics

Table 1 presents the sociodemographic characteristics of the sample by sexual orientation. Compared to heterosexuals, the proportion of women and younger individuals was higher among sexual minority individuals. Sexual minority individuals had lower income and lower education compared to heterosexuals and were less often employed. Around one third of sexual minority individuals lived with a partner, compared to more than half of heterosexuals. Most of the sample lived in urban areas and was born in Sweden.

**Table 1:** Sociodemographic characteristics of the sample (young adults in Sweden, 2019, *N* = 2, 126) by sexual orientation

	Heterosexuals (81.3%)	Sexual minority individuals (18.7%)	*p*-value^1^
	%	%	
Gender			
Male Female Other gender identity	44.1 55.9 0.0	26.2 67.9 5.9	**< 0.001**
Age groups			
18–24 25–34	37.0 63.0	47.9 52.1	**< 0.001**
Educational level			
High school or less Some college/ technical school University degree	53.1 11.5 35.4	57.9 15.9 26.2	**< 0.001**
Employment status			
Not employed Employed	26.5 73.5	40.9 59.1	**< 0.001**
Income			
< 10 000 SEK^2^/month 10 000–29 999 SEK/month ≥ 30 000 SEK/month	24.4 48.3 27.3	40.3 44.3 15.4	**< 0.001**
Relationship status			
Not living with a partner Living with a partner	47.0 53.0	65.5 34.5	**< 0.001**
Urbanicity			
Urban area Rural area	84.1 15.9	83.1 16.9	0.601
Country of birth			
Sweden Other	89.2 10.8	90.5 9.5	0.477

^1^*p*-value is based on a χ-test or Fisher’s exact test; bold indicates *p* < 0.05 ^2^ Swedish kronor

### Sexual orientation differences in mental health services use and unmet mental health care needs

A larger proportion of sexual minority individuals (35.0%) reported having used mental health services during the previous 12 months (Table 2) compared to heterosexuals (20.2%). The regression model adjusted for sociodemographic covariates showed that sexual minority individuals were 52% (OR = 1.52, 95% CI: 1.17–1.96, *p* = 0.002) more likely than heterosexuals to report having used mental health services during the previous 12 months. Sexual minority individuals were also significantly more likely to report unmet mental health care needs (17.6%) compared to heterosexuals (11.8%, OR = 1.47, 95% CI = 1.09-2.00, *p* = 0.013). In the subgroup analysis with only those participants perceiving a need for mental health services, there was no significant difference in unmet mental health care needs by sexual orientation.

**Table 2:** Odds ratio for sexual orientation differences in use of mental health services and unmet mental health care needs (young adults in Sweden, 2019, *N* = 2,126)

*USE OF MENTAL HEALTH SERVICES*							
	%	cOR^1^	95% CI^2^	*p*-value	aOR^3^	95% CI^2^	*p*-value
Sexual orientation							
Heterosexual	20.2	1			1		
Sexual minority	35.0	2.13	1.68–2.69	< 0.001	1.52	1.17–1.96	0.002
*UNMET MENTAL HEALTH CARE NEEDS*							
	%	cOR^1^	95% CI^2^	*p*-value	aOR^3^	95% CI^2^	*p*-value
Sexual orientation							
Heterosexual	11.8	1			`1		
Sexual minority	17.6	1.59	1.18–2.12	0.002	1.47	1.09-2.00	0.013
*UNMET MENTAL HEALTH CARE NEEDS (AMONG THOSE WITH PERCEIVED NEED)*							
	%	cOR^1^	95% CI^2^	*p*-value	aOR^3^	95% CI^2^	*p*-value
Sexual orientation							
Heterosexual	37.0	1			1		
Sexual minority	33.5	0.86	0.61–1.19	0.353	1.00	0.70–1.43	0.998

^1^ cOR: Crude odds ratio ^2^ CI: Confidence intervals ^3^ aOR: Adjusted odds ratio (adjusted for gender, age, educational level, employment status, income, relationship status, urbanicity and country of birth)

### Sociodemographic differences in mental health services use and unmet mental health care needs by sexual orientation

**Fig. 1:**
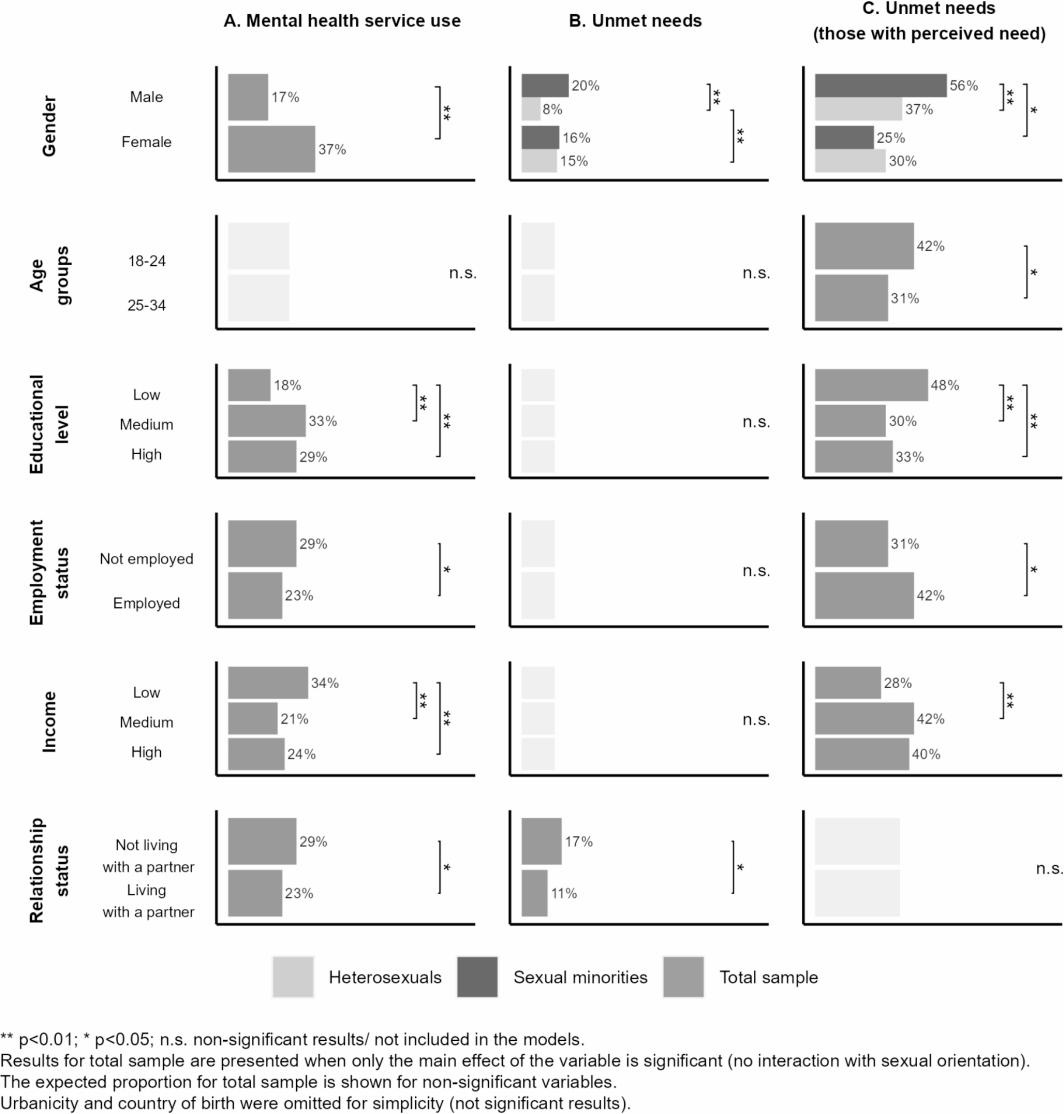
Expected proportions of mental health services use and unmet needs by sociodemographic factors (young adults in Sweden, 2019, *N* = 2, 126)

In addition to sexual orientation differences, mental health services use differed by gender, educational level, employment status, income, and relationship status. However, none of the interactions between these variables and sexual orientation were significant. Sociodemographic differences in mental health services use are presented as expected proportions in Fig. 1-A and the complete results of the logistic regression model are presented in Online Resource 2 - Table S1. Mental health services use was more common among females compared to males, those not employed, those not living with a partner, and those with low income. Those with a low level of education were less likely to use mental health services compared to those with higher level of education.

Figure 1-B presents the expected proportions for unmet mental health care needs by sociodemographic variables (among the total sample), with the complete results of the logistic regression models being presented in Online Resource 2 - Table S2. It shows a higher proportion of unmet mental health care needs among females (vs. males) and those not living with a partner (vs. those living with a partner). Moreover, there was a significant interaction between gender and sexual orientation. Heterosexual men (8%) were less likely to report unmet mental health care needs than sexual minority men (20%) and heterosexual women (15%). There was no significant sexual orientation difference in unmet mental health care needs among women.

In the subgroup analysis including only those with a perceived need for mental health services, unmet mental health care needs differed by gender, age group, educational level, employment status, and income. In this subsample of individuals who perceived a need for mental health services, analyses showed a higher proportion of unmet mental health care needs among younger participants (18–24 years), employed participants, and participants with low education compared to older participants (25–34 years), unemployed participants, and participants with medium and high educational level. In this subsample, participants with low income were less likely to have unmet mental health care needs than those with medium income. There was a significant interaction between gender and sexual orientation and the likelihood of not receiving mental health treatment among those with a perceived need for such services. An estimated 56% of sexual minority males reporting a need for mental health treatment had not received such services, compared to 37% of heterosexual males and 25% of sexual minority women. There was no sexual orientation difference in the likelihood of not receiving mental health treatment among women with a perceived need for such services. The sociodemographic difference in unmet mental health care needs and the interaction effect for gender by sexual orientation are presented as expected frequencies in Fig. 1-C; Online Resource 2 – Table S3 presents the complete results of the logistic regression model.

## Discussion

Sexual minority individuals’ disproportional greater risk of common mental health problems is one of the most salient and enduring disparities in psychiatric epidemiology [1, 2, 5]. Whether this increased risk of problems corresponds to a similarly increased mental health services use, or if the expected higher need for mental health care among sexual minorities is more likely to be unmet, has lacked sufficient evidence to date [7, 19]. This study takes advantage of a large probability-based sample to compare mental health services use and unmet mental health care needs between sexual minority and heterosexual young adults. Results showed that both sexual minority men and sexual minority women were more likely to use mental health services than their heterosexual counterparts. Although, overall, sexual minorities were more likely to experience unmet mental health care need compared to heterosexuals, subsequent subgroup analyses showed that this disparity was only significant among men. Among women, there was no sexual orientation difference in unmet mental health care needs.

Results of this study confirm previous findings showing a higher likelihood of mental health services use among sexual minority individuals compared to heterosexuals. It is one of the first studies to report this pattern in a European context using a representative population-based sample [1, 8, 19]. In our sample, women were more likely to use mental health services, and there was no interaction between gender and sexual orientation. Our results suggest that the increased likelihood of using mental health services among women exists for both heterosexuals and sexual minority individuals. This finding is contrary to a previous study from the US showing that the gender gap in mental health services use that exists among heterosexuals, in which women use more mental health services than men, was not present among sexual minorities [8, 28]. Similar to what has been found in previous studies, the likelihood of using mental health services differed by education, income, employment, and relationship status, with the same pattern existing for both sexual minority individuals and heterosexuals.

Our study showed that unmet mental health care needs were more common among sexual minority men compared to heterosexual men, indicating that one in five sexual minority young men perceive a need for mental health treatment without receiving such services. This represents a public health concern, since living with untreated mental health disorders has been shown to influence the development of more severe diseases [26]. Further, the increased risk of unmet mental health care needs among sexual minority individuals was equally distributed by most sociodemographic factors, but somewhat higher among those not living with a partner. This is consistent with previous evidence that being married or cohabiting with someone is associated with better mental health [29, 30].

While unmet mental health care needs did not significantly differ by sexual orientation among those with a perceived need for care, gender was a moderator of these differences. More than half of sexual minority young men perceiving a need for mental health treatment did not receive such services, which was a significantly higher proportion than among heterosexual men and sexual minority women. Research into the utilization of mental health services by sexual minority men is limited [8] and insufficient to provide a clear explanation of these results. Future research should use populational-based samples to study how barriers to mental health services are unequally distributed by gender and sexual orientation. Our results did not show any sexual orientation differences in unmet mental health care needs among women with a perceived need for care. The role of gender as a moderator of sexual orientation difference in unmet mental health care needs has been described before in one US study; however, in that study heterosexual women were more likely to have unmet mental health care needs than sexual minority women [15].

Among those with perceived need for mental health services, previous studies have shown that some groups seem less likely to seek help. For example, some studies have shown that young adults avoid seeking mental health care because they feel embarrassed or lack previous experiences of help-seeking [31, 32]. Those with low levels of education have been shown to be less likely to attribute their symptoms to a mental disorder, to have more negative attitudes towards mental health treatment and to be less aware about where they can get access to help [33–35]. People who are employed may have less daytime availability to seek professional care [36], while individuals with a disability may be more likely to use health services, to manage their health, or to ask for sick leave [36], and students may use services available in colleges. Previous research has shown an association between low income and barriers related to service accessibility [33]; however, some studies with population-based samples have not confirmed income-related inequalities in unmet mental health care needs [37]. Unmet mental health care needs may strongly depend on one’s inability to pay for services, and persons with low-income can access them in primary health care in an affordable manner.

In this study, other than gender, there were no significant interactions between demographic factors and sexual minority status in predicting mental health service use or unmet mental health care needs. This indicates that the sociodemographic factors linked to higher mental health services use (e.g., low income, unemployment, higher education) have a similar impact on sexual minority and heterosexual individuals. The lack of interactions between demographic factors and sexual minority status can possibly be due to Sweden having relatively low degree of structural discrimination towards LGBT individuals and relatively accepting population attitudes towards homosexuality [38], wherein sexual minority status may not constitute an additional disadvantage to socioeconomic inequalities. The high proportion of sexual minority men perceiving a need for mental health treatment who do not receive such services suggests a need for future studies identifying the determinants and barriers impacting this group’s use of mental health services.

In this study, around one-third of those with perceived need for mental health services did not receive them, consistent with previous evidence in the Swedish population [20]. Understanding the experiences of sexual minority individuals when accessing mental health services is important to decrease this gap, particularly among sexual minority men, who seem more likely to have unmet mental health care needs than heterosexual males and sexual minority women. Making mental health services more accessible and affirmative of the needs of sexual minority individuals may improve help-seeking behaviors among sexual minority men. LGBTQ + networks and organizations can have a pivotal role in this, through the communication of previous positive experiences of mental health services among sexual minority individuals [39], or the provision of services free of discrimination [40]. Use of internet-based services may also help to overcome some barriers when accessing mental health services [40]. Additionally, considering that sexual minority individuals are more likely to use mental health services than heterosexuals, efforts should be undertaken to boost their quality of care and to equip mental health professionals to deal with sexual minority individuals’ distinct experiences and mental health needs [41, 42], including by assessing minority stress and treating its mental health sequelae.

A major strength of the present study is its use of a representative sample of young adults, thus enhancing generalizability of results [7]. Yet, while we used sampling weights to correct for non-response, some sort of selection bias may remain, with overrepresentation of individuals with heightened awareness of mental health issues. However, due to sample size concerns, sexual minority subgroups were collapsed under one dichotomized variable, thus hindering the understanding of differences between non-heterosexual subgroups, including those affecting bisexual individuals [7, 43]. Particularly, future research should study whether use of mental health services differ between heterosexuals, gay/lesbian, bisexual and individuals from less studied sexual minority subgroups (e.g., demisexual and asexual). In this study, participants’ need for mental health services was self-reported and we do not know how well such self-reports correspond to clinically assessed mental health symptoms and diagnosis. Previous research suggests that this correspondence may be stronger among heterosexual than sexual minority individuals [15].

Overall, the results of this study show that, compared to heterosexual young adults, sexual minority young adults are more likely to use mental health services, stressing the need to tackle determinants of mental health among this population and to invest in effective, culturally sensitive mental health services. Yet, unmet mental health care needs were common, particularly among sexual minority men, whose determinants and mediators of help-seeking behaviors demand further research.

## Electronic supplementary material

Below is the link to the electronic supplementary material.

Supplementary Material 1

Supplementary Material 2

## Data Availability

The statistical code is available from the corresponding author. Under Swedish law and ethical approval, individual level data of this kind cannot be publicly available. Individual level data can be made available on reasonable request as long as it is in line with Swedish law and ethical approvals.
