# Supplementary figures and images for: Sexual orientation differences in mental health service use and unmet mental health care needs: a cross-sectional population-based study of young adults
Source: Soc Psychiatry Psychiatr Epidemiol. 2025 Mar 11;61(2):225–34. doi: 10.1007/s00127-025-02866-8 (PMC12948820; doi:10.1007/s00127-025-02866-8)

## Online Resource 1

### Flowchart with the sample selection

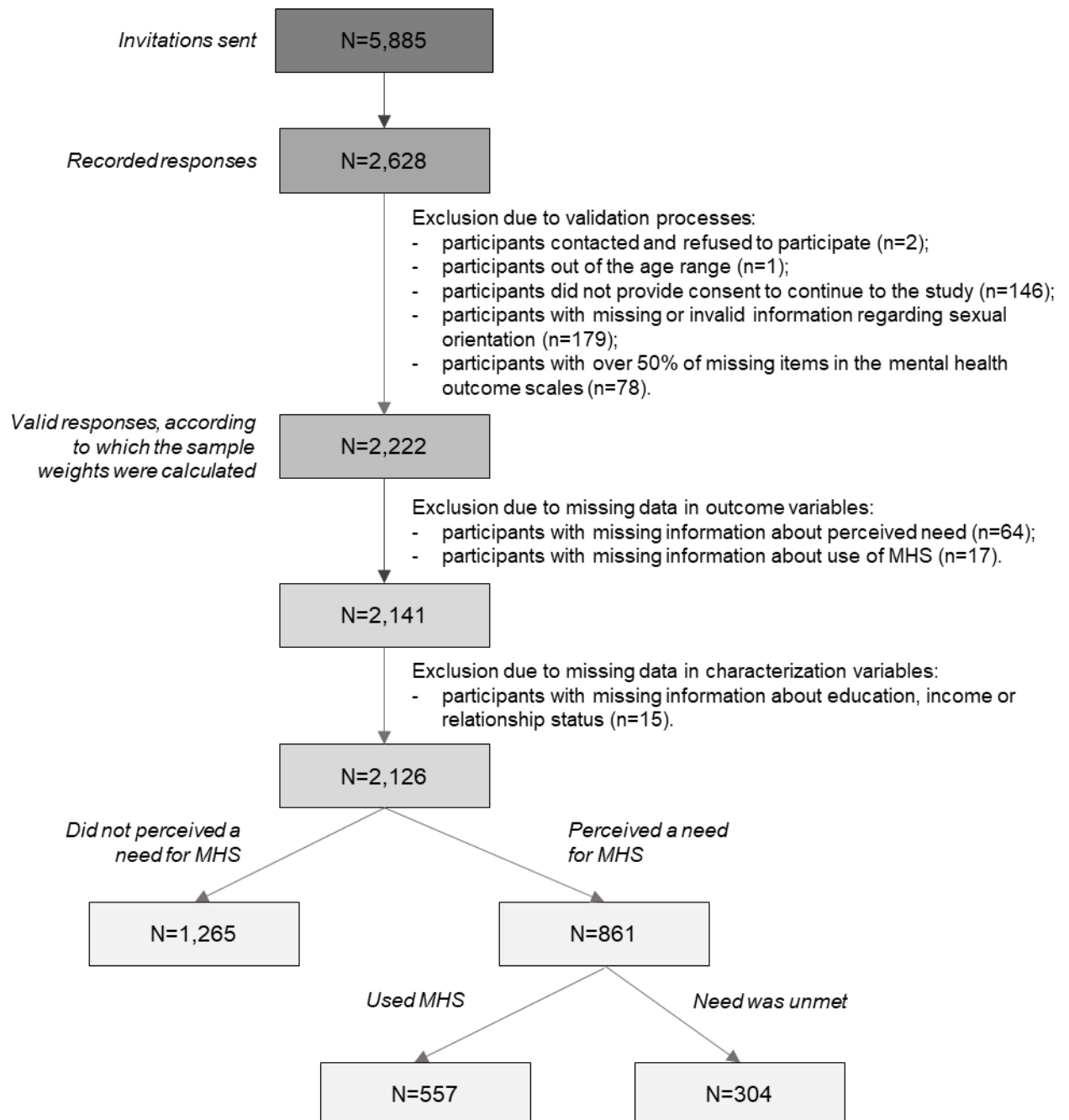

Supplement: Supplementary file 1 — Supplementary Material 1 [file 127_2025_2866_MOESM1_ESM.pdf]
