# Supplementary material for: Sexual orientation differences in mental health service use and unmet mental health care needs: a cross-sectional population-based study of young adults
Source: Soc Psychiatry Psychiatr Epidemiol. 2025 Mar 11;61(2):225–34. doi: 10.1007/s00127-025-02866-8 (PMC12948820; doi:10.1007/s00127-025-02866-8)
Supplement: Supplementary file 2 — Supplementary Material 2 [file 127_2025_2866_MOESM2_ESM.pdf]

Online Resource 2 – Table S1

Bivariate and multivariate logistic regression models for mental health services use by sexual orientation and sociodemographic factors (N=2,085)

	Bivariate analyses of main effects for each sociodemographic factor			*Separate analyses of main effect for each sociodemographic factor, sexual orientation, and interaction effects*			*Multivariate analyses including all sociodemographic variables and all significant interactions*		
	OR[1]	95% CI[2]	*p*-value[3]	OR[1]	95% CI[2]	*p*-value[3]	OR[1]	95% CI[2]	*p*-value[3]
Sexual orientation									
Heterosexual	1						1		
Sexual minority	2.00	1.57-2.55	**<0.001**				1.55	1.20-2.00	**<0.001**
Gender									
Male	1			1			1		
Female	3.02	2.39-3.81	**<0.001**	2.70	2.09-3.50	**<0.001**	2.90	2.27-3.70	**<0.001**
Female * SM				1.39	0.74-2.62	0.304			
Age groups									
17-24	1			1					
25-34	1.06	0.86-1.31	0.586	1.18	0.92-1.51	0.185			
25-34 * SM				0.78	0.48-1.28	0.325			
Educational level									
High school or less	1			1			1		
Some college/ technical school	1.76	1.30-2.38	**<0.001**	1.91	1.33-2.72	**<0.001**	2.33	1.67-3.23	**<0.001**
University degree	1.40	1.12-1.75	**0.003**	1.69	1.31-2.17	**<0.001**	1.93	1.46-2.55	**<0.001**
Some college * SM				0.67	0.34-1.32	0.248			
University degree * SM				0.50	0.29-0.88	**0.017**			
Employment status									
Not employed	1			1			1		
Employed	0.63	0.51-0.78	**<0.001**	0.67	0.52-0.86	**0.002**	0.75	0.58-0.97	**0.026**
Employed * SM				0.98	0.59-1.61	0.931			
Income									
<10 000 SEK[4]/month	1			1			1		
10 000 - 29 999 SEK/month	0.51	0.41-0.65	**<0.001**	0.51	0.39-0.67	**<0.001**	0.53	0.40-0.70	**<0.001**
≥30 000 SEK/month	0.56	0.43-0.73	**<0.001**	0.60	0.44-0.82	**<0.001**	0.61	0.42-0.88	**0.008**
10 000 - 29 999 * SM				1.23	0.72-2.11	0.452			
≥30 000 SEK/month * SM				1.00	0.49-2.03	0.994			
Relationship status									
Not living with a partner	1			1			1		
Living with a partner	0.77	0.63-0.95	**0.012**	0.83	0.66-1.04	0.107	0.75	0.59-0.94	**0.015**
Living with a partner * SM				1.04	0.63-1.73	0.871			
Urbanicity									
Urban	1			1					
Rural	1.19	0.91-1.55	0.210	1.19	0.88-1.62	0.263			
Rural * SM				0.96	0.51-1.82	0.902			
Country of birth									
Sweden	1								
Other	0.75	0.53-1.06	0.101	0.78	0.53-1.16	0.223			
Other * SM				0.84	0.36-2.00	0.697			

[1] OR: Odds Ratio [2] CI: Confidence intervals [3] Bold indicates p<0.05 [4] Swedish kronor

Online Resource 2 – Table S2

Bivariate and multivariate logistic regression models for unmet mental health needs by sexual orientation and sociodemographic factors (N=2,085)

	Bivariate analyses of main effects for each sociodemographic factor			*Separate analyses of main effect for each sociodemographic factor, sexual orientation, and interaction effects*			*Multivariate analyses including all sociodemographic variables and all significant interactions*		
	OR[1]	95% CI[2]	*p*-value[3]	OR[1]	95% CI[2]	*p*-value[3]	OR[1]	95% CI[2]	*p*-value[3]
Sexual orientation									
Heterosexual	1						1		
Sexual minority	1.66	1.23-2.23	**<0.001**				2.84	1.69-4.79	**<0.001**
Gender									
Male	1			1			1		
Female	1.54	1.18-2.00	**0.002**	1.81	1.33-2.46	**<0.001**	1.92	1.41-2.62	**<0.001**
Female * SM				0.37	0.20-0.70	**0.002**	0.38	0.20-0.71	**0.002**
Age groups									
17-24	1			1					
25-34	0.67	0.52-0.86	**0.002**	0.62	0.46-0.82	**0.001**			
25-34 * SM				1.61	0.89-2.92	0.115			
Educational level									
High school or less	1			1					
Some college/ technical school	0.75	0.50-1.14	0.179	0.67	0.40-1.10	0.114			
University degree	0.73	0.55-0.97	**0.028**	0.70	0.51-0.96	**0.027**			
Some college * SM				1.39	0.57-3.38	0.471			
University degree * SM				1.38	0.69-2.74	0.359			
Employment status									
Not employed	1			1					
Employed	1.01	0.77-1.34	0.934	1.14	0.82-1.59	0.446			
Employed * SM				0.80	0.43-1.48	0.470			
Income									
<10 000 SEK[4]/month	1			1					
10 000 - 29 999 SEK/month	0.92	0.69-1.23	0.574	0.94	0.67-1.32	0.704			
≥30 000 SEK/month	0.67	0.47-0.96	**0.027**	0.63	0.42-0.96	**0.030**			
10 000 - 29 999 * SM				1.07	0.55-2.07	0.854			
≥30 000 SEK/month * SM				1.87	0.80-4.42	0.151			
Relationship status									
Not living with a partner	1			1			1		
Living with a partner	0.63	0.48-0.81	**<0.001**	0.59	0.44-0.79	**<0.001**	0.62	0.48-0.81	**<0.001**
Living with a partner * SM				1.64	0.88-3.04	0.119			
Urbanicity									
Urban	1			1					
Rural	0.88	0.62-1.25	0.471	0.83	0.55-1.26	0.376			
Rural * SM				1.22	0.55-2.73	0.626			
Country of birth									
Sweden	1			1					
Other	0.77	0.50-1.20	0.255	0.68	0.40-1.15	0.150			
Other * SM				1.73	0.64-4.66	0.277			

[1] OR: Odds Ratio [2] CI: Confidence intervals [3] Bold indicates p<0.05 [4] Swedish kronor

Online Resource 3 – Table S3

Bivariate and multivariate logistic regression models for unmet mental health needs among those with perceived need for mental health care by sexual orientation and sociodemographic factors (N=835)

	Bivariate analyses of main effects for each sociodemographic factor			*Separate analyses of main effect for each sociodemographic factor, sexual orientation, and interaction effects.*			*Multivariate analyses including all sociodemographic variables and all significant interactions.*		
	OR[1]	95% CI[2]	*p*-value[3]	OR[1]	95% CI[2]	*p*-value[3]	OR[1]	95% CI[2]	*p*-value[3]
Sexual orientation									
Heterosexual	1						1		
Sexual minority	0.92	0.66-1.29	0.642				2.21	1.07-4.53	**0.031**
Gender									
Male	1			1			1		
Female	0.60	0.43-0.84	**0.002**	0.75	0.52-1.10	0.138	0.75	0.51-1.10	0.141
Female * SM				0.37	0.16-0.82	**0.014**	0.35	0.15-0.80	**0.013**
Age groups									
17-24	1			1			1		
25-34	0.68	0.50-0.91	**0.010**	0.57	0.40-0.81	**0.002**	0.63	0.43-0.93	**0.021**
25-34 * SM				1.83	0.93-3.61	0.080			
Educational level									
High school or less	1			1			1		
Some college/ technical school	0.51	0.32-0.81	**0.004**	0.42	0.24-0.74	**0.003**	0.46	0.28-0.76	**0.003**
University degree	0.58	0.42-0.81	**0.001**	0.48	0.33-0.70	**<0.001**	0.53	0.34-0.81	**0.004**
Some college * SM				1.91	0.71-5.14	0.197			
University degree * SM				2.27	1.03-4.98	**0.042**			
Employment status									
Not employed	1			1			1		
Employed	1.44	1.04-1.97	**0.026**	1.53	1.04-2.25	**0.030**	1.63	1.12-2.37	**0.011**
Employed * SM				0.79	0.39-1.60	0.515			
Income									
<10 000 SEK[4]/month	1			1			1		
10 000 - 29 999 SEK/month	1.54	1.10-2.16	**0.012**	1.60	1.07-2.38	**0.022**	1.84	1.23-2.74	**0.003**
≥30 000 SEK/month	1.08	0.72-1.64	0.707	0.98	0.61-1.57	0.920	1.67	0.95-2.94	0.074
10 000 - 29 999 * SM				0.85	0.40-1.80	0.666			
≥30 000 SEK/month * SM				1.64	0.61-4.36	0.324			
Relationship status									
Not living with a partner	1			1					
Living with a partner	0.81	0.60-1.09	0.169	0.73	0.52-1.03	0.076			
Living with a partner * SM				1.47	0.72-2.98	0.287			
Urbanicity									
Urban	1			1					
Rural	0.78	0.52-1.17	0.237	0.74	0.46-1.19	0.210			
Rural * SM				1.25	0.51-3.10	0.624			
Country of birth									
Sweden	1			1					
Other	1.01	0.60-1.70	0.976	0.87	0.47-1.61	0.647			
Other * SM				1.77	0.55-5.72	0.341			

[1] OR: Odds Ratio [2] CI: Confidence intervals [3] Bold indicates p<0.05 [4] Swedish kronor
